# Modern Surgery

**Published:** 1861-04

**Authors:** 


### Art. VI.—MODERN SURGERY.

It must surely appear strange to some of the older men in our profession to look back upon the practice of early years, to note what advances have been made within the space of a single lifetime, to observe how the strongly expressed opinions of some successful surgeon or physician have exercised for a time a dominant influence over many departments of professional knowledge, and how statements and facts, once thought irrefragable, have been extended and modified or altogether overthrown by increasing experience and wider knowledge. Not that the great principles of medical science are ever fundamentally changed. The aphorisms of Hippocrates contain the germs of much that later times have only more fully developed. And it would not be difficult for a good pathologist of the present day to reconcile many of the conflicting opinions, and settle the acrimonious disputes of the solidists and humoralists of the last century. In many instances, like the shield between the knights of old, the same fact was only different because looked at from different sides, and were we now to revert to their wordy contests, let us hope that, like the fox in the fable, we should be able to extract the oyster and leave the old combatants the empty shell of disputation to divide between them.

Yet, though it be admitted that the fundamental doctrines of our science do not materially change from age to age, yet the precise method in which these doctrines are to be applied to the treatment of any particular disease requires constant modification according to circumstances. Hence the value of experience. Were it not so, the young man, fresh from the schools, familiar with anatomy and physiology, and learned in all that books can teach him of medicine and surgery, would be trusted in more by reason of this knowledge. Yet the old man maintains, as he probably ever will maintain, his position of superiority, because the doctrines that we teach or learn are always empirical; because the application of medicine or surgery can never be made a matter of rule; because the practised hand and eye, the cool head, the calm judgment of mature age are constantly required, and it is but seldom that in any of these the young can contend with the old.

As in the wide field of microscopic research, it is not that we now require a higher magnifying power, but rather the careful and diligent application of the powers we possess to the elucidation of the difficult and obscure; so in surgery and medicine, it is not that we require more means, but rather that the means and knowledge we have already obtained should be well applied. Our arms must not only be bright, polished, and fit for use, but we should have the skill on occasion dexterously and skilfully to wield them.

The time is yet far distant, it perhaps may never arrive, when every organ whose structure has been seriously impaired either by disease or accident, or whose function is materially disordered, will be certainly and completely restored by medical treatment. Yet so many instances of recovery from various forms of disease in many of the organs of the body might be adduced, that we can scarcely help believing that all are equally amenable to treatment, if we possessed only the clue which might guide our practice. That clue is undoubtedly to be derived from a threefold source: from a careful investigation of healthy structure, from a close observation of the causes and results of disease (ætiology and pathology), and, lastly, from assiduous and careful examination of the effects of remedies. How necessary a consideration of all these circumstances is may be shown by a reference to a disease over which we have a very complete control—iritis. Iritis is an affection attacking one of the most delicate muscular and vascular structures with which we are acquainted, running a rapid course, accompanied by severe pain, generally resulting, if left alone, in the entire abolition of sight, and in irremediable alteration of structure. But we happen now to hold the clue. We know that opium will relieve the pain; that calomel will, as Dr. Billing believes, prevent the effusion, or, as others suppose, promote the absorption, of lymph. We administer them together, and, though not absolutely essential in the treatment, we have a powerful auxiliary in a remedy which acts on the muscular tissue—belladonna. The pupil being maintained fully dilated by its aid, the value of which we only thoroughly recognize by a minute anatomical examination of the structures implicated, success of the happiest and most complete kind crowns our efforts; perfect vision is at once the brilliant antithesis of total blindness, and the desired result of skilfully directed knowledge.

We might almost repeat the above remarks in speaking of valvular disease of the heart, a masterly exposition of the proper methods and value of treatment in which, is given by Dr. Latham, in his Clinical Lectures, in a mode that must be familiar to all professional readers.

So with enlargement of the spleen in ague, with such affections as croup and diphtheria, which, though often fatal, yet are to a great extent in their early stages, and to some extent even in their later stages, under our control. In these instances we can, at least, see that we are in the right path ; but there are many others where the light we possess to guide is of the feeblest kind, and these appear to be chiefly in the blood diseases. What, for example, is our knowledge of the deadly remittents of Africa and of the West India Isles; what of plague, of cholera, what even of pyæmia, the too frequent consequence of many operations ?

We cannot but admit that in the last few years, during which continually increasing attention has been paid to pathology, many minor improvements have been made in various departments of surgery, as well as of medicine, consisting for the most part in closely following the operations of nature in her happiest efforts. These improvements collectively assume such consequence that we have thought them worthy of a brief *résumé*.

The importance of preventing the access of air to a cut surface has, it appears to us, been only fully recognised during a comparatively short period. Yet isolated examples of what would now be called subcutaneous surgery are met with in the ancient operation of depression of cataract as performed by the Greeks, and in such operations as that for the relief of hernia by Petit. Formerly (indeed, we believe the practice exists at the present day in some of the foreign hospitals), all open wounds were filled with scraped lint, or charpie, and allowed slowly to heal by granulation. It mattered not whether they were made by the clean instruments of the surgeon, or were lacerated, or contused in their character, the treatment was the same. When the great tedium and loss of time by this method were recognised by the medical man, as they had long been by the patient, the next step made in advance was in the immediate closure of clean incised wounds, the abolition of poultices, and the substitution for these last of simple water dressing. This change was in great measure due, in England, to its advocacy by Mr Liston, the success of whose practice was so great that the plan, simple, natural, and cleanly as it was, rapidly spread through the country.

But stitches of silk and plaster were still often used, and their presence setting up much irritation, prevented union in their immediate neighbourhood, and it often occurred that, in removing the plaster, the tender and newly-formed adhesions were torn asunder. Yet more recently, the introduction of the metallic suture, the use of a fine and perfectly smooth silver wire instead of silk, which possesses the advantage of setting up little or no irritation in the tissues through which it passes, has been followed by marked benefit. There was yet one point of difficulty and annoyance. The ligatures with which, in any large operation, the various arteries were tied, these forming in many instances a thick bundle, still kept the edges of the wound apart and retarded recovery. Even this difficulty has, however, been surmounted by the ingenuity of Dr. Simpson, who has suggested that hæmorrhage from arteries, even of the largest size, may be controlled by pressure upon the vessels, made by thrusting a long needle through the textures. By these several means we appear to have reached at length the ultimatum of success in the treatment of simple incised wounds. There is now no irritating substance whatever between the lips of the wound, there is nothing, consequently, to hinder, if the constitution be healthy, union by the first intention; and we accordingly find that large wounds, such as those produced by the removal of the breast in females, of a large tumour, or even by the amputation of a limb, may be completely healed in a few days.

There can be no doubt of the immense advantage which attends the performance of operations of whatever kind subcutaneously. The brilliant success of such operations has been well stated in an oration by Mr. Adams, read before the Medical Society of London in 1857, and he has collected a very large number of instances in which the principles of subcutaneous surgery are carried out. Thus, besides the ordinary operations for club-foot and for strabismus, which are now almost uniformly performed subcutaneously, he mentions Sir B. Brodie's plan of division of varicose veins beneath the skin, the opening of bursa, and of many abscesses, especially of those which occur in scrofulous children, of buboes, as recommended by Mr. Milton, and the operation for the radical cure of hernia. In all these instances the knife is small. It is introduced through sound skin; and the wound, which is necessarily minute, is carefully closed with a small wad, kept applied by a light bandage. Ill consequences are almost unknown, and the healing of the divided textures progresses with wonderful rapidity, and is accomplished by the development of cells, which form a fibrous connecting tissue. The good results of this plan are so generally recognised, that it may be said with truth to be uniformly adopted whenever the case will admit of it. This, then, may be considered as one of the most important, because one of the most generally applicable improvements of modern surgery.

The now generally adopted treatment of fractures may be considered as another improvement of modern surgery. Time was, when the unfortunate man who had fractured his arm or leg suffered severely at the hands of his medical attendant, and some of the plans suggested are so entirely opposed to the spirit of modern surgery, that we can only feel surprise at the conservative power of nature, which out of means so imperfect, could yet produce favourable results.

In a book scarcely yet out of date, written by a retired naval officer, who refers with pride to the capital results of his treatment in numerous instances, it is stringently ruled that the man with a fractured thigh should be allowed to rest in an easy position for a week before any attempt be made to help him, and that *then* the limb should be extended, and be packed, *secundum artem,* in splints, that is to say, exactly at the period when, according to modern research, nature is rousing herself to activity in the formation of new bone, the parts, by handling and twisting, are to be reduced to their original condition at the time of the accident. The simplicity of the modern apparatus for the treatment of fracture is specially worthy of remark. Straight splints, a swing fracture-box for fractures of the leg, and occasionally a double inclined plane for fractures of the thigh, with a few bandages, are almost all that are required, and in many cases even these, simple as they are, are superseded by the application of the plaster of Paris or white of egg bandage. The use of these enables the patient to move about much earlier, by ensuring accurate coaptation, and does away with the long and tedious confinement which was formerly thought necessary. The simplest plan of treatment we have ever heard of for any fracture is that described by the late Mr. Vincent in cases of fractured clavicle, which he proposes to treat by simple position, and, from our own experience, we can most cordially recommend it, the patient being placed upon a hard mattress upon his back, so that the weight of the shoulders may open the chest and bring the fractured ends into position. If the patient can maintain this position for three weeks, sufficient union will have occurred to enable him to get up, only taking the precaution to keep the arm in a sling, that it may not be involuntarily used.

It may almost be said that the principles of subcutaneous surgery are brought into practice in the treatment of cases of compound fracture. Here the surgeon hastens to reduce the bone, if protruding, to close and heal the wound in the soft parts, to convert the injury, as far as possible, to the conditions of a simple fracture; and though, of course, in accidents of such serious import as these failure often occurs, yet sufficient encouragement is given by their occasional recovery to indicate to us that we are following the dictates of nature. At all events we are sure of this, that more instances of this kind recover now than would have been the case in the early part of this century, when the general character of surgical proceedings was of a more meddlesome nature, and when the restorative powers of life were less confidently relied on.

No single discovery in the science of medicine ever led to such important results as the application of chloroform for the prevention and relief of pain. That various substances have been used for these purposes at periods long antecedent to our era is well known. Operations of a painful nature were long ago performed under the influence of wine, of the extract of hemp, and of the juice of some of the Solanaceæ. The inhalation of various gases was suggested towards the close of the last century; those employed, however, were found to be inefficient in practice, and their use was soon abandoned. In 1846 an American surgeon, Morton, used the vapour of ether for the purpose of producing insensibility, with success, and in a few months it was everywhere tried. Its disagreeable odour, the violent cough produced by it, and the occurrence of one or two fatal accidents, led chemists to turn their attention to other volatile fluids. The employment of chloroform we owe to the sagacity, and it may also be said to the courage, of Dr. J. Y. Simpson, of Edinburgh, who, after a long series of experiments, fortunately chanced to meet, in chloroform, with a substance of equal anæsthetic value to ether, and far more pleasant in operation; and although occasionally serious results have followed its use, these have generally been traceable to neglect of proper precautions; and, on the whole, in practised hands, it may be said to be perfectly safe.

Still more recently the application of freezing mixtures has been suggested, to parts that are painful or which are about to be operated on. In some cases, great advantage results from this local or topical application of a benumbing agent; but it is only in comparatively few instances that it can be adopted.

Chloroform, undoubtedly, is the means which, more than any other, has enabled the surgeons of the present day to advance to so extraordinary a degree in the path of conservative surgery, and nowhere is this more conspicuous than in the treatment of diseased joints. Whilst the patient is insensible, the surgeon can ascertain the amount of motion retained by the joint; he can determine in some measure the extent of the disease; he can introduce probes; he may even make exploratory incisions; and if he find that the cartilage or extremities of the bone are alone affected, he can quietly and without interruption proceed to the removal of the articulating surfaces. He need not be an old man who can recall instances where limbs have been (what we should now call) fairly sacrificed to these painful and protracted diseases; instances which, if they now fell into the hands of a good surgeon, would certainly be operated upon for resection. In many of these cases the movements of the hand and fingers, or of the ankle joints, have been perfect. Yet, because some old standing inflammation or ulceration of the elbow or knee joints existed, the arm or thigh has over and over again been amputated, leaving only a useless stump. Now, by freely laying open the joint, and the removal of two small fragments (a daring yet singularly successful operation) a hand and foot have often been left, which to the outward eye differed not at all, and to the patient himself but little from the opposite and healthy limb. In some of the admirably successful cases of Mr. Jones, of Jersey, which we have had an opportunity of seeing, no lameness at all was visible, when the patients wore high-heeled boots. It is curious that these operations, involving as they do long and deep incisions with much care upon the part of the operator, rarely require any vessels to be tied: upon the whole, their results appear to be superior to those of ordinary amputations.

In ophthalmic surgery the great achievement of the last few years has been the discovery of the ophthalmoscope. By its means many diseases, formerly classed together in a confused manner, under the general terms of "amaurosis" and "glaucoma," are now easily distinguished from one another, and, as a natural consequence, the treatment which was formerly comprised in a few very general rules, is now directed to definite objects, and is far more successful. As in most other discoveries, the principle has long been known; its application has alone been wanting. The principle may be shown simply in this way,—when we look into a dark cellar at the farther end of which some animal, as a cat, is gazing directly at us, their eyes assume a singular glaring or illuminated appearance. It is due to the reflection from the bottom of their eyes of the light entering at the door behind us. If we are situated obliquely, as regards the animal, or if it is looking aside, no illumination is seen; the reflected light is stopped by the margin of the iris. In examining the human eye, therefore, it is necessary that the light should be thrown into it from the same point as that from which we are looking. This is accomplished by a small concave mirror having a minute hole in the middle through which the observer looks; a lamp is placed behind the patient; a ray of light can thus be easily thrown directly into his eye. A convex lens of about two inches focus is found to be essential to the thorough investigation of the bottom of the eye. Many cases, the nature of which would formerly have been exceedingly obscure can now be determined with the utmost certainty; thus, incipient cataract, detachment of portions of pigment, haziness of the vitreous humour, congestion or anæmia of the retinal vessels, and atrophy of the choroid or deposit of lymph upon its surface, are all affections the nature of which can be very satisfactorily ascertained by ophthalmoscopic investigation, and many can be materially benefitted by treatment. Even glaucoma, so long a stumbling-block to the ophthalmic surgeon, bids fair to yield to the long and patient investigation to which it has been subjected. By the older surgeons it was regarded as almost hopeless. Lawrence recommends purgatives, bleeding, and mercurials. The elder Guthrie used to apply a leech every night for a month to the temple. Then, paracentesis, or tapping, was tried; but all these plans fell into disuse. At length Gräfe, drawing his conclusions partly from anatomical and pathological facts, and partly from symptoms disclosed by the ophthalmoscope, as cupping of the optic nerve, enlargement and change of form and pulsation in the retinal vessels, suggested an operation which has now been frequently tried with varying success; but which is probably, with some slight modifications, destined to hold a high place amongst the curative measures to be employed for this insidious and destructive disease. His operation consists essentially in the removal of a portion of the iris.

Mr. Hancock, taking another view of the pathology of glaucoma, has recommended another operation which has been repeated with immaterial modifications by Nurreley and Solomon—namely, the division of the ciliary muscle, the good effects of which in some of the cases we have seen and operated on have been indubitable; but the whole subject is still too fresh for any positive statement to be made. The treatment of another series of affections of the eye has materially improved of late years. In purulent ophthalmia, formerly, the most severe anti-inflammatory treatment, both local and general, was employed, and but too often with unfavourable results; now, the patients are supported, good food, wine, and quinine are given instead of abstinence and bloodletting, and the eyes kept clean by emollient, or but slightly stimulant lotions, instead of being burnt with caustics, and, rapid as the progress of the disease often is, good vision is very generally preserved.

Such are a few of the chief improvements in modern surgery; many more might be added were our space not limited. Much of this general advance is no doubt due to men who, possessing wide knowledge in every department of professional information, have devoted their whole energies to the cultivation of some particular branch. It is to such devotion that we owe many of our best works—such works as *Brodie on the Joints*, *Lawrence on Hernia*, and *Mackenzie on the Diseases of the Eye*; but we do, in common with the authors of the *Protest against Special Hospitals*, which has been recently extensively circulated, most strongly deprecate the limitation of the younger members of the profession to the pursuit of some particular form of disease, merely to gain a name, their general information on other branches being weak and imperfect.

In a handicraft trade, subdivision of labour commonly implies improvement in the manufacture of each part; but in knowledge it is not so. The wider the grasp, the stronger the powers of the intellect; by so much the more easily will it handle any subject presented to it for the first time. Complete knowledge of a profession is essential to the prosecution with advantage of any special part. A man who shall engage in the practice of one disease without a competent knowledge of the nature and causes of disease in general, will almost certainly fall into a routine plan of treatment; he will be unable to see the bearings upon broad questions, which oftentimes the most trivial facts possess, and, on the other hand, will almost as certainly be unable to perceive how the progressive knowledge of other departments is associated with, and intimately related to, the advance of his own particular branch. We would make it a rule that every surgeon to a special hospital should also be a surgeon or assistant-surgeon to a general hospital.

Coincident with the progress of general and scientific knowledge has been the advancement of surgery. During the last hundred, we might almost say during the last fifty years, more real improvements have been made than during the five hundred which preceded them. The theory of the art has been made more exact, its practice more perfect; the objects to be attained have been more clearly perceived—the means of accomplishing them more thoroughly understood. Instead of being the master, the surgeon is content that his art should be the handmaid of nature; he is willing in most cases to follow in her footsteps, content if occasionally he may interpose to direct or modify the processes of reparation which she ever strives to establish.

---

